# Visualization of neonatal lung injury associated with mechanical ventilation using x-ray dark-field radiography

**DOI:** 10.1038/srep24269

**Published:** 2016-04-13

**Authors:** Andre Yaroshenko, Tina Pritzke, Markus Koschlig, Nona Kamgari, Konstantin Willer, Lukas Gromann, Sigrid Auweter, Katharina Hellbach, Maximilian Reiser, Oliver Eickelberg, Franz Pfeiffer, Anne Hilgendorff

**Affiliations:** 1 Lehrstuhl für Biomedizinische Physik, Physik-Department & Institut für Medizintechnik, Technische Universität München, Garching, Germany; 2 Comprehensive Pneumology Center, Helmholtz Zentrum Muenchen, Munich, Germany, Member of the German Center for Lung Research (DZL); 3 Institute for Clinical Radiology, Ludwig-Maximilians-University Hospital Munich, Munich; 4 Department of Neonatology, Perinatal Center, Dr. von Haunersches Children’s Hospital, Ludwig-Maximilians University, Munich, Germany

## Abstract

Mechanical ventilation (MV) and supplementation of oxygen-enriched gas, often needed in postnatal resuscitation procedures, are known to be main risk factors for impaired pulmonary development in the preterm and term neonates. Unfortunately, current imaging modalities lack in sensitivity for the detection of early stage lung injury. The present study reports a new imaging approach for diagnosis and staging of early lung injury induced by MV and hyperoxia in neonatal mice. The imaging method is based on the Talbot-Lau x-ray grating interferometry that makes it possible to quantify the x-ray small-angle scattering on the air-tissue interfaces. This so-called dark-field signal revealed increasing loss of x-ray small-angle scattering when comparing images of neonatal mice undergoing hyperoxia and MV-O_2_ with animals kept at room air. The changes in the dark field correlated well with histologic findings and provided superior differentiation than conventional x-ray imaging and lung function testing. The results suggest that x-ray dark-field radiography is a sensitive tool for assessing structural changes in the developing lung. In the future, with further technical developments x-ray dark-field imaging could be an important tool for earlier diagnosis and sensitive monitoring of lung injury in neonates requiring postnatal oxygen or ventilator therapy.

Early lung injury in the neonatal lung is often provoked by oxygen supplementation or mechanical ventilation (MV) or both (MV-O_2_), established as critical life-saving treatment strategies in postnatal care. Due to the immature morphology of the lung, these treatment options are also known to induce defective alveolar septation, impaired angiogenesis and pathologic extracellular matrix remodeling resulting in lung growth impairment^1^,^2^,^3^. In the long term, these changes result in neonatal chronic lung disease (nCLD), also known as Bronchopulmonary Dysplasia (BPD)^4^, frequently complicating the course of preterm or risk term birth. Along with asthma and cystic fibrosis, nCLD is one of the most common chronic lung diseases in children, whose incidence is reported to be as high as 77% in neonates born at less than 32 weeks of gestation^5^,^6^. Although outgrowing oxygen dependency by the age of two years, many infants with BPD have episodes of wheezing, require inhalation therapies^7^ or show indications of poor pulmonary gas transfer and significantly lower peak workload at school age^8^. In consequence, adolescent nCLD patients show impaired pulmonary function including a reduction in FEV_1_ that may be regarded as a precursor of COPD at an older age^9^.

Due to the severity of the disorder and the associated treatment costs, there is an urgent need for a diagnostic tool to early and reliably detect, stage and monitor morphological changes associated with lung injury caused by mechanical ventilation and oxygen toxicity. Current clinical routine is based on the use of conventional x-ray chest radiography^10^,^11^,^12^, which is limited by low sensitivity for the detection of pulmonary morphological changes^11^. CT has been demonstrated to provide much more meaningful results^13^,^14^, yet its use is severely limited due to the associated high radiation exposure of the infants. Access to lung function information is limited in the clinical setting especially after cessation of invasive ventilation. Spirometric tests that largely depend on patients’ compliance have been shown to suffer from a high variability^15^,^16^ and do not yield spatial information.

Developing x-ray imaging further, a method has been recently reported that makes it possible to acquire additionally to conventional x-ray absorption information, x-ray phase-contrast and dark-field signals^17^,^18^,^19^. Thereby, information about the small-angle x-ray scattering registered in the dark field^19^,^20^ has been shown to significantly increase lung tissue visibility on radiographic images in mice^21^ and to improve the detection of calcifications in mammographic scans^22^. The acquisition of this imaging modality is based on the introduction of a three-grating Talbot-Lau interferometer into the x-ray beam. The change in refractive index between tissue and air causes x-rays to be refracted on each air-tissue interface in the lung, resulting in small-angle scattering. Thus, a strong dark-field signal has been observed for healthy lungs in mice^21^. Moreover, it could be demonstrated that detection of changes to the lung structure can be significantly improved based on dark field compared to absorption x-ray imaging as shown by the analysis of pulmonary emphysema^23^,^24^,^25^,^26^ and fibrosis^27^ in mice.

Bringing together the need for a sensitive diagnostic tool in neonatal chronic lung disease and the novel technique of x-ray dark-field imaging in this study, we evaluated for the first time the capability of grating-based x-ray dark-field radiography to detect very early changes in morphology of the neonatal developing lung undergoing injury induced by hyperoxia and MV using a unique, pre-clinical mouse model^3^,^28^,^29^,^30^,^31^.

## Results

### Detection of morphological changes in the neonatal lung undergoing MV-O_2_ using x-ray dark-field radiography

**Figure 1:**
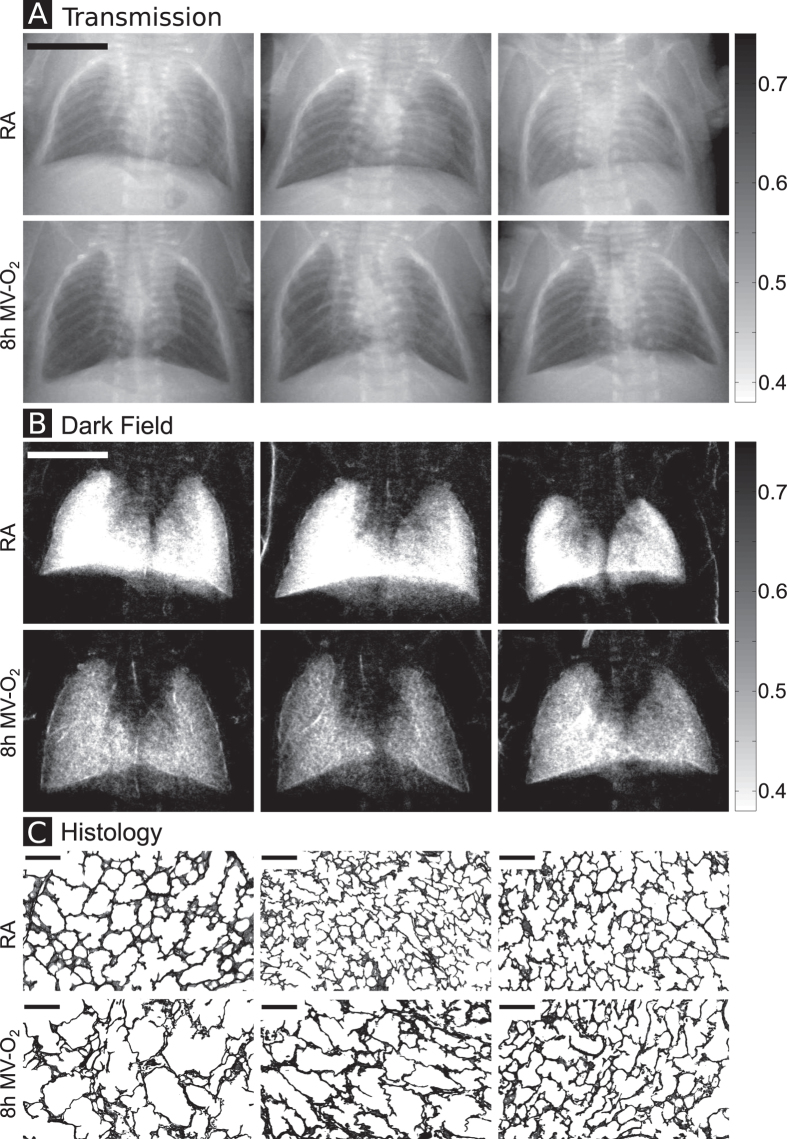
Contrary to conventional x-ray transmission (**A**) that reveals only hardly appreciable differences, dark-field (**B**) radiograms yield a clear discrimination in terms of signal intensity between the lungs from animals in the room air (RA) group as compared to neonatal mice undergoing MV-O_2_ for 8 h. (**C**) The corresponding histological sections. Scale bars for radiographies correspond to 5 mm and for histology to 100 μm.

**Figure 2:**
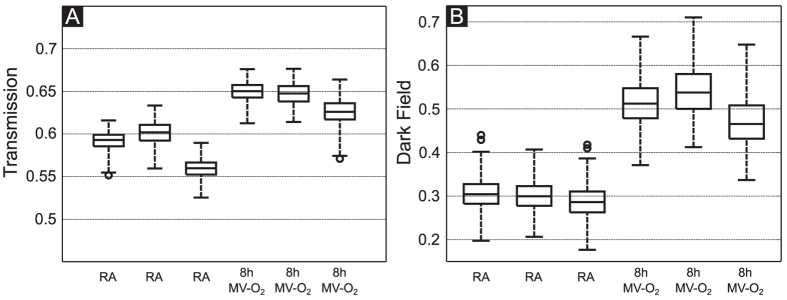
Boxplots showing the distribution of (**A**) transmission and (**B**) dark-field signal for the six mice shown in Fig. 1.

As shown in Fig. 1, x-ray dark-field radiograms, in contrast to conventional x-ray transmission projections, were able to visualize changes in the neonatal mouse lung induced by MV-O_2_. Unventilated lungs from animals breathing room air appear bright, whereas the diseased lungs display a reduced dark-field signal intensity, due to reduction in air-tissue interfaces. Histology supported the dark-field imaging results with larger and fewer alveoli in the lungs of neonatal mice undergoing MV-O_2_ (Fig. 1(C)). Dark-field radiograms furthermore showed an increase in signal heterogeneity in neonatal mice undergoing 8 hours of MV-O2 (Fig. 2), which, apart from the overall signal decay, reflects inhomogeneity in lung injury and consecutive remodeling.

### Sensitivity of imaging to different degrees of neonatal lung damage

**Figure 3:**
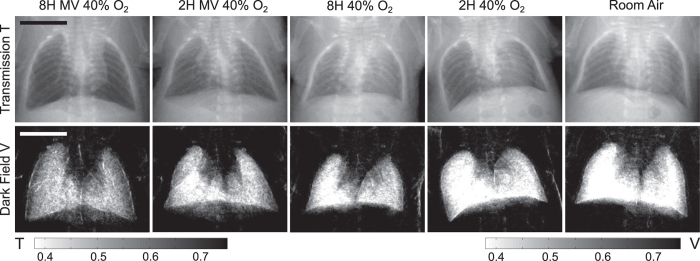
Transmission (top row) and dark-field (bottom row) radiograms of animals undergoing moderate hyperoxia (fiO_2_ 0.4) or MV-O_2_ for 2 and 8 h. Whereas images obtained with dark-field technology (top row) show a stepwise decrease in signal and an increase in scatter inhomogeneity, transmission images (bottom row) do not allow for sufficient discrimination between the groups. Scale bars correspond to 5 mm.

To assess the sensitivity of the imaging method with respect to early changes, a series of transmission and dark-field radiographies were performed in mice that were exposed to moderate hyperoxia for 2 or 8 hours or to short (2 h) or medium length (8 h) MV-O_2_, respectively. Results were compared to images of mice breathing room air. As presented in Fig. 3, the dark-field signal intensity showed a stepwise decrease when comparing the lungs from neonatal mice exposed to hyperoxia for 8 h and to MV-O_2_ for 2 and 8 h with respect to the animals from the room air group. In contrast, the transmission radiograms did not allow a reliable discrimination of the groups.

**Figure 4:**
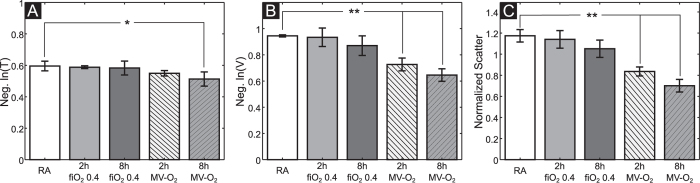
Signal quantification shows moderate changes in transmission signal (**A**), where as analysis of dark field (**B**), and normalized scatter (**C**) visualizes a stepwise decrease in signal intensity when comparing animals undergoing hyperoxia and MV-O_2_ with mice breathing room air. The error bars represent the group SDs. n(RA) = 3; n(2 h fiO_2 0.4) = 3; n(8h fi O_2 0.4) = 4; n(2h MV-O_2) = 6; n(8h MV-O2) = 5; *p < 0.05, **p < 0.01.

Quantification of the imaging signal intensities (Fig. 4) confirmed these observations. Whereas analysis of the transmission signal revealed only modest differences when comparing the different experimental groups, x-ray dark-field radiography yielded a significantly improved discrimination between the animal groups. Quantification of transmission images showed a maximum change of 14% in logarithmic transmission values, when comparing images obtained from animals after 8 h of MV-O_2_ with results from mice breathing room air. In contrast, analysis of the dark-field radiograms revealed a stepwise scattering decrease, reaching significance as early as in the group of animals undergoing 2 h of MV-O_2_. The maximum logarithmic signal difference of 32% was observed between the groups of 8 h MV-O_2_ and room air controls. A similar clear discrimination result was obtained also for the normalized scatter, with a maximum difference of 40% between the room air control animals and 8 h MV-O_2_ mice.

### Histologic assessment of structural changes to the neonatal lung and pulmonary tests

**Figure 5:**
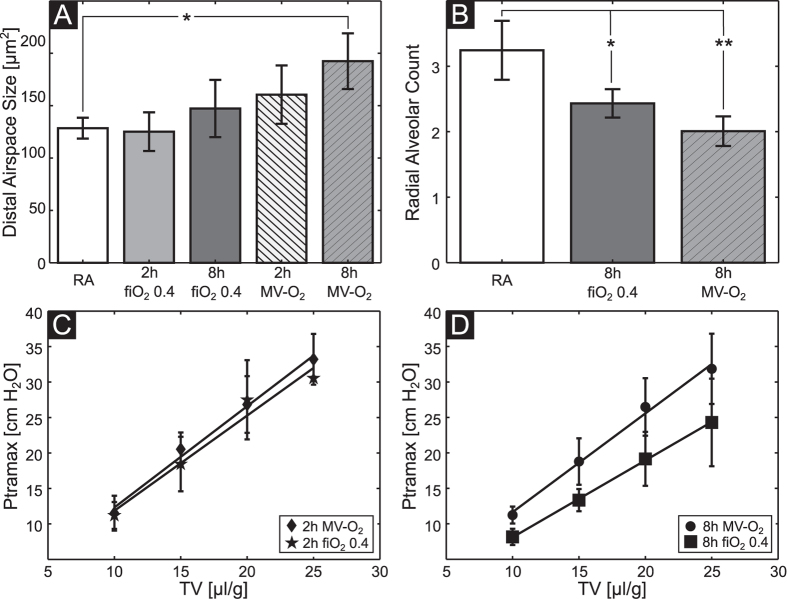
Quantitative histologic assessment shows a step-wise increase in distal airspace size (**A**) and a decrease in RACs (**B**) when comparing the lungs from animals breathing room air with mice undergoing hyperoxia and MV-O_2_. (**C**) and (**D**) visualize the mean dynamic compliance and standard deviation as a function of tidal volume TV. The error bars represent the group SDs. n(RA) = 3; n(2 h fiO_2_ 0.4) = 3; n(8 h fiO_2_ 0.4) = 4; n(2 h MV-O_2_) = 6; n(8 h MV-O_2_) = 5; *p < 0.05, **p < 0.01.

Histologic analysis showed a step-wise increase in distal airspace size and a decrease in radial alveolar counts (RAC) reaching significance when comparing lungs from mice breathing room air with animals undergoing 8 h of MV-O_2_ (Fig. 5). Quantification of RACs reached a significant difference when comparing lungs from animals after moderate hyperoxia for 8 h and pups breathing room air. Lung volumes measured by fluid displacement before histology were found to not differ significantly between the groups, e.g. RA: 53.9 ± 3.4 μl/g; 8 h MV-O_2_: 56.2 ± 3.1 μl/g. Percentage of atelectasis was not significantly different between the groups and did not exceed 30% in relation to total lung tissue.

Lung function analysis, comprising assessment of tidal volume and airway pressure by whole body plethysmography only revealed differences when comparing animals undergoing 8 h of MV-O2 with their respective hyperoxia controls (Fig. 5).

### Correlation of imaging with histological parameters

**Figure 6:**
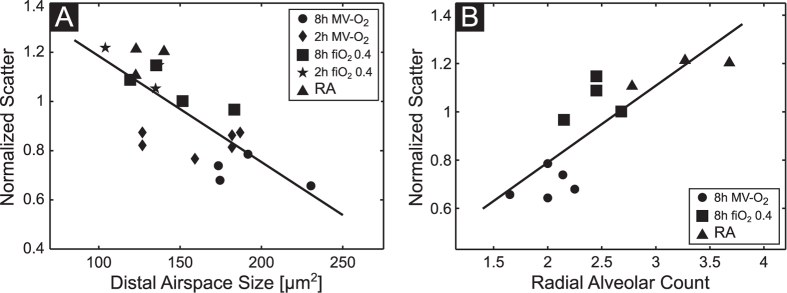
Distal airspace size (**A**) and RACs (**B**) show a linear correlation with the normalized scatter (Pearson’s correlation coefficient -0.75 (**A**) and 0.82 (**B**)).

Distal airspace size and alveolar number, estimated by radial alveolar counts show a linear correlation with the normalized scatter (Fig. 6) with a Pearson’s correlation coefficient of −0.75 (distal airspaces size) and 0.82 (RAC), respectively. In contrast, the correlation coefficient with the transmission signal was only −0.67 (distal airspace size) and 0.76 (RAC), respectively.

## Discussion

In the present study we have demonstrated that grating-based x-ray dark-field radiography can be used to significantly improve the visualization of early changes in the neonatal developing mouse lung undergoing MV-O_2_. The transmission signal is not sensitive enough to visualize the changes in airspace size and quantity. In contrast, x-ray dark-field radiography is picking up the architectural changes in the lung, thereby providing valuable visualization of early pulmonary changes and allowing for the assessment of different degrees of lung injury. As diffracted x-ray photons are detected in the same detector pixel without influencing the transmission signal in the absence of the grating interferometer, the result highlights the complementarity of the x-ray transmission and the dark-field imaging technique. Only the x-ray dark-field signal is able to pick up the small diffraction angles, thereby generating sensitive information about the pulmonary structure, i.e. airspace enlargement. In order to account for the inter-animal variability, a combination of x-ray dark-field and conventional transmission imaging, referred to as normalized scatter, was calculated. The normalized scatter reflects the pulmonary architectural changes induced by increase in airspace size and loss of (septal) tissue and reveals the best differentiation between the different experimental groups. Due to the developmental stage of the murine lung, the airspace can refer to alveoli themselves or the alveolar duct as secondary septation is still ongoing.

Imaging results obtained by dark-field radiography were related to quantitative histological analysis. With respect to the clinical relevance of the model, histological analysis displays changes to the lung structure that mimics the characteristics of pulmonary injury observed in infants undergoing MV-O_2_. This has been confirmed in previous studies^32^.

The early pulmonary changes in the lung undergoing postnatal injury can be attributed to an increase in i) apoptosis or ii) extracellular matrix remodeling with the resulting airspace enlargement and interstitial remodeling reflected by the signal alteration in dark-field imaging. The detection of these early pulmonary changes, long-term potentially leading to BPD development, is of uttermost importance to allow for adequate and individualized treatment strategies in the mechanically ventilated neonate. In contrast, pulmonary function tests show a lower sensitivity for the detection of early morphological changes and only differentiate for the changes observed in neonatal mice that underwent 8 h of MV-O_2_. This result furthermore emphasizes the necessity for a more sensitive, advanced diagnostic approach in the clinical setting, where conventional x-ray techniques and lung function analysis serve as the main diagnostic tools.

The results obtained by the current study will open a new perspective for future management of neonates that require mechanical ventilation with oxygen enriched gas and are at a high risk to develop long-term changes to the pulmonary architecture. X-ray dark-field imaging allows for the detection of early detrimental effects by MV-O_2_ in neonates and can thereby help to monitor treatment approaches including the development of differentiated ventilation protocols protecting the developing lung from injury. To overcome the limitation of 2D imaging of superimposition of ventral and dorsal lung regions, the additional acquisition of lateral radiograms have to be considered.

Although X-ray dark-field radiography is still a preclinical technique, a recent study reported the development of a system that has a field of view that is suitable for imaging the thorax of an infant^33^ and showed the feasibility of acquiring x-ray dark field radiograms at a clinically compatible dose^33^. In mice, the dose used to acquire the small-animal radiographies is estimated with 1.4 mGy, so that longitudinal *in vivo* small-animal measurements can be performed without restrictions^24^,^25^. In order to obtain x-ray transmission and dark-field images, a precise movement of one of the gratings by a fraction of its period is required between the acquisitions of a minimum of two images. As the grating periods are typically in the range of a few μm, a high precision for the movement of the grating is mandatory. As this prerequisite can be challenging for clinical applications, several algorithms have been recently developed to adjust for the impact of imprecise grating movement^34^. Nonetheless, the technical implementation of x-ray grating interferometer into clinical imaging systems will require additional optimization.

The reasoning for an *ex vivo* imaging approach was based on two important technical challenges that needed to be addressed. On the one hand, the differing degree of pulmonary inflation induced by the process of MV led to the implementation of standardized inflation volumes immediately after the disconnection from the ventilator in order to allow for the assessment of structural changes by avoiding the artificial impact of differences in inflation levels. On the other hand, the optimization of the experimental conditions in neonatal mice ventilation required the reduction of dead space and optimization of temperature conditions, which could not be achieved inside the scanner.

Nonetheless, future research should focus on applying x-ray dark-field imaging in parallel to the ventilation procedure in order to investigate changes in the lung while undergoing MV-O_2_. Furthermore, these studies should take the spatial information for the signal analysis into account and seek to identify the most affected areas of the lung.

## Materials and Methods

### Neonatal mouse ventilation

Animal experiments were performed with permission of the Institutional Animal Care and Use Committee of the Helmholtz Zentrum Munich and carried out in accordance with national (Gesellschaft für Versuchstierkunde - Society for Laboratory Animal Science) and international (Federation for Laboratory Animal Science Associations) animal welfare guidelines. All experimental protocols were approved by the Institutional Animal Care and Use Committee of the Helmholtz Zentrum Muenchen, Munich, Germany. The study was carried out in 5–7-day-old C57B6 wild type mice born at term gestation (3.2 ± 0.4 g). Neonatal mice were assigned to the following groups: unventilated animals spontaneously breathing 40% O_2_ for 2 or 8 hours or room air (21% O_2_) (n = 3–6). Randomly chosen littermates received mechanical ventilation with oxygen-enriched gas (MV-O_2_; 40% O_2_) for 2 or 8 hours (n = 4–6). For MV-O_2_, pups received a tracheotomy under sedation with subcutaneous ketamine (~60 μg/g body weight, bw) and xylazine (~12 μg/g bw), before transfer to MV at 180 breaths/min with a customized, small animal respirator (MicroVent 848; Harvard Apparatus, Holliston, MA). The ventilation settings mimic clinical protocols with a volume-guaranteed strategy applying mean tidal volumes of 8.68 μl/g bw, stepwise decreased after the onset of MV, and airway pressures of 12–13 cmH_2_O (peak) and 11–12 cmH_2_O (mean)^35^. All unventilated animals received sham surgery under mild sedation with ketamine and xylazine.

### Pulmonary Function Tests

In a subset of animals, tidal volume and airway pressure (maximum tracheal pressure, Ptramax) measurements were performed using whole body plethysmography (Pulmodyn, Hugo Sachs Elektronik, Harvard Apparatus GmbH, Hugstetten, Germany). As the impact of the chest wall can be neglected in the newborn, tidal volume of the ventilator was increased in a stepwise manner from 10 to 25 μl/g bw to assess the quasi-static compliance of the respiratory system. The animals were allowed to relax for 60 seconds between different tidal volumes which were applied for 30 seconds.

### Imaging Protocol

In order to obtain x-ray transmission and dark-field images it is necessary to acquire a minimum of two images with a different relative position of the gratings in the grating interferometer. Therefore, one of the gratings is typically moved by a fraction of its period between the images, thereby achieving the possibility to discriminate between x-ray absorption and the scattering processes. From the acquired original images the intrinsically perfectly registered transmission and dark-field images were calculated using the Fourier decomposition^18^. Imaging was performed with a prototype small-animal x-ray phase-contrast and dark-field scanner^36^,^37^. On the ventilator, pups were euthanized with sodium pentobarbital, lungs were filled with a standardized volume of 150 μl room air via the intra-tracheal tubing and animals were transferred immediately to the dark-field scanner. *Ex vivo* imaging was chosen in order to i) standardize inflation volume to optimize quantification of structural changes of the lung and to ii) meet critical requirements for MV in neonatal mice including the reduction of dead space and temperature optimization. The scanner was operated at 35 kVp (source 17 W), acquiring images for 5 different source grating positions, with an exposure time of 3.3 seconds per image. The sample resolution was approximately 60 μm (10% MTF). The grating interferometer consisted of a gold source grating (period p = 10 μm, height h = 35 μm), a nickel phase grating (p = 3.24 μm, h = 4 μm) and a gold analyzer grating (p = 4.8, h = 45 μm). The distances between the source to the phase and the analyzer grating are 30 and 45 cm, respectively.

### Quantitative Morphometry

After imaging, all lungs were harvested for histological analysis through intra-tracheal fixation with buffered 4% paraformaldehyde at a standard pressure of 20 cm H_2_O as previously reported^32^. After fixation, lung volume was measured using fluid displacement^38^. Lungs were subsequently embedded in paraffin. Histologic analysis was performed blinded to the animal’s group assignment and included airspace size, radial alveolar counts (RAC) and septal counts. As published previously, these measures allow estimating airspace enlargement together with a reduction in alveolar/airspace number in the case of unchanged lung volumes as measured by fluid displacement. The percentage of atelectasis in relation to total lung tissue was quantified using the ImageJ software. Measurements of alveolar area, i.e. airspace size was performed in 4 high power fields per tissue section in 2–3 independent random tissue sections (4 μm, H&E) per animal (CAST-Grid 2.1.5; Olympus, Ballerup, Denmark). A median of ≥30 fields of view in 2–3 random tissue sections per animal was used to estimate alveolar number by the use of radial alveolar counts (RAC) as described previously^39^.

### Image Analysis

The imaging signals were quantified blinded to the animal’s group assignment using a manually created mask identical for x-ray transmission and dark-field radiograms, as both imaging modalities are intrinsically perfectly registered. The mask was chosen to fit as much lung tissue as possible, omitting the heart shadow. As the ribs are not fully developed in the pups and are barely visible on the transmission images, their influence was neglected. The signal intensities were subsequently analyzed using Matlab R2013b (MathWorks, Natick, MA, USA). Since the dark field scales exponentially with the sample thickness^20^,^40^ similar to the Beer-Lambert law for transmission imaging, the natural logarithm of both transmission T and dark field V were quantified.

In order to get rid of the sample thickness dependence, associated with projection-based imaging, a ratio of the transmission and dark field was calculated, referred to as normalized scatter N^41^. The normalized scatter is obtained as:


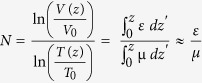


where V_0_ and T_0_ are the dark field and transmission amplitudes in front of the sample and ε and μ are the diffusion and attenuation coefficient, respectively.

### Statistical analysis

Data are given as mean and standard deviation (SD). Statistical analysis was performed using Matlab R2013b (MathWorks, Natick, MA, USA). The datasets were compared using Student’s unpaired t-test or the non-parametric Mann-Whitney test for results with skewed distribution when two groups were compared. For the comparison of three or more groups the N-Way ANOVA was applied with respective post-hoc tests as appropriate. Differences were considered statistically significant when the p value was <0.05.

## Additional Information

**How to cite this article**: Yaroshenko, A. *et al.* Visualization of neonatal lung injury associated with mechanical ventilation using x-ray dark-field radiography. *Sci. Rep.* **6**, 24269; doi: 10.1038/srep24269 (2016).
